# Sensory substitution and augmentation techniques in cerebral visual impairment: a discussion of lived experiences

**DOI:** 10.3389/fnhum.2025.1510771

**Published:** 2025-02-10

**Authors:** Stephanie L. Duesing, Katie Lane-Karnas, Sebastian James Adam Duesing, Mae Lane-Karnas, Nai Y, Arvind Chandna

**Affiliations:** ^1^Independent Researcher, Naperville, IL, United States; ^2^Independent Researcher, Calais, VT, United States; ^3^Independent Researcher, Chicago, IL, United States; ^4^Independent Researcher, San Francisco, CA, United States; ^5^Smith-Kettlewell Eye Research Institute, San Francisco, CA, United States; ^6^Alder Hey Children’s Hospital, Liverpool, United Kingdom

**Keywords:** CVI, sensory substitution, cerebral visual impairment, O&M, sensory augmentation, braille, orientation and mobility, verbal mediation

## Abstract

Pediatric vision loss due to cerebral visual impairment (CVI) is an urgent public health issue, demanding evidence-based (re)habilitation and educational strategies. As with other neurodiverse populations, research on CVI needs to be directly informed by the lived experiences of those affected—children, adults, and their families. In this paper, three individuals with early-onset CVI and two parents discuss sensory substitution and augmentation developed in childhood in the absence of early identification of CVI, and they detail the important impact of the empowering, professionally taught non-visual skills–such as braille, orientation and mobility training, and assistive technology–which were acquired later. Efforts to improve visual perception ability and understanding of the visual world, both effective and ineffective, were made through traditional, professionally administered vision therapy, self-taught coping strategies, and from intensive arts participation. The authors discuss the strategies they use to leverage senses other than vision to achieve their daily life, educational, social, and career goals. Nonvisual skills training effective in those with ocular blindness, though received later in life after the delayed diagnosis, proved to be indispensable for these authors’ who have CVI access to all aspects of independent life. It is our hope that these personal experiences may encourage research into how traditional nonvisual skills training used for the ocularly blind, as well as sensory substitution and augmentation techniques, may be used to develop evidence-based multidisciplinary interventions; improved academic and independent life skills; multisensory educational and therapeutic interventions; and successful integration into the community for all CVIers.

## Introduction

Cerebral visual impairment (CVI) is the number one cause of severe visual impairment/blindness in children in higher income countries (Solebo et al., 2017; Chong and Dai, 2014; Williams et al., 2021; West et al., 2021; Teoh et al., 2021; Kessel et al., 2024), and the prevalence is increasing worldwide (Pehere et al., 2019; Aghaji et al., 2015; Ozturk et al., 2016). CVI can result from several different causes but commonly occurs due to adverse events in the prenatal or perinatal period, which lead to brain injury affecting processing of vision. CVI is characterized by challenges with visual recognition and visually guided behaviors of action – also termed higher visual function deficits or visual perception deficits (HVFDs; VPDs) and variable loss of visual acuity and visual field defects (Fazzi et al., 2007; Philip and Dutton, 2014; Boot et al., 2010; Zihl and Dutton, 2015; Chandna et al., 2021a, 2021b, 2024). HVFDs can impact those affected so significantly that their CVI informed clinician reports their functional vision is at the level of legal blindness, even in the case of typical VA (Kran et al., 2019). The definition and scope of the term “blindness” has been the subject of debate for over a century with recommendations from as early as 1893 (Elementary Education [Blind and Deaf Children] Act) to extend it beyond visual acuity and visual field to include “too blind” to perform everyday activities and a special dispensation for those “too blind to be able to read the ordinary school books” (Smith and Paton, 1915); a critique of visual acuity as a sole measure of blindness (Harman, 1926; Ray et al., 2016); the limited inter-relationship between visual function and functional vision (Colenbrander, 2009) and recent debates on criteria of blindness specific to CVI (Kran et al., 2019; McConnell et al., 2021; Pilling et al., 2023). In this paper, we incorporate the National Federation of the Blind’s functional definition, where the term” blind “refers to anyone who must devise alternative techniques to do anything which he would do with sight if he had normal vision regardless of the cause of the vision loss (Jernigan 1984, 1995).

CVI is poorly understood, often underdiagnosed, diagnosed late and, at worse, misdiagnosed because severity of vision impairment continues to be defined by VA, which may be normal in CVI (van Genderen et al., 2012; Chandna et al., 2021a, 2021b, 2024). In addition, most clinicians and low vision professionals lack awareness of CVI (Maitreya et al., 2018; Pilling, 2022; Oliver et al., 2023). This ignorance is made worse by a lack of reliable tools in routine clinical practice to assess HVFDs though such tools are being evaluated in research (Philip and Dutton, 2014; Lueck et al., 2019; McConnell et al., 2021). Referral for (re)habilitation is historically based on low VA and reduced visual field, and outcomes are typically measured by VA (World Health Organization (WHO), 2022). Parents may notice symptoms but are hard pressed to find low vision professionals who have medically and scientifically accurate knowledge of CVI, and who can tease out what symptoms are due to CVI and what symptoms are due to other conditions. According to the National Institutes of Health (NIH), because CVI often coexists with various medical conditions and learning disabilities, it can be difficult to determine which specific condition is causing a patient’s difficulties and this challenge highlights the importance of prioritizing CVI in the diagnostic process (Chang et al., 2024; Gordon et al., 2024). In the US, more than 155,980 young people who have CVI are currently undiagnosed (CVI Center, 2023) and living without non-visual skills training (Appendix), making access to a CVI informed, multidisciplinary team crucial.

Prior to identification of CVI, those who have the condition are unaware of their visual perceptual problems as they did not develop the higher visual functions and are therefore unaware that neurotypical, sighted people have those abilities (Goldenberg et al., 1995; Heilman, 1991; Celesia and Brigell, 2005). This results in underdiagnosis and, worse, a misdiagnosis (Lueck et al., 2019; Chokron and Dutton, 2023), with one in thirty mainstream-educated children having at least one CVI-related vision problem (Williams et al., 2021).

The critical importance of listening to and understanding patient experiences has been reported for visual impairment (VI) (Douglas, 2006; Robertson et al., 2021; Lupón et al., 2018). Qualitative interviews investigating CVI families’ experiences have emphasized the importance of more work in this area (Jackel et al., 2010; Goodenough et al., 2021; Oliver et al., 2023). Understanding the lived experiences of CVIers who have awareness of the challenges with HVFDs and who have developed successful multisensory strategies to deal with HVFDs in CVI is paramount for understanding of the condition; clinical and research direction; habilitation planning; and to provide context for families making decisions for their children.

In this paper, two adults (Sebastian Duesing, Nai) and one teenager (Mae Lane-Karnas) who have CVI, two parents (Stephanie Duesing, Katie Lane-Karnas), and their clinical partner (Arvind Chandna) discuss what VPDs exist and how the authors who have CVI independently and non-consciously developed sensory substitution and augmentation techniques to learn and engage in daily life. These coping strategies for learning and surviving as blind people in a visual world, without any appropriate non-visual skills training, were developed in early childhood, in two cases through intensive participation in the arts. Before medical diagnosis of CVI, Duesing, Lane-Karnas, and Nai all experienced ineffective outcomes in regard to improving CVI symptoms with efforts to improve functional vision in vision therapy. These three authors all later pursued training in and use non-visual skills in daily living. Lane-Karnas and Nai struggled with reading print and their challenges are described and discussed in each section.

## Diagnosis of CVI

Though it is now recognized that CVI typically has an early onset due to adverse events at or around time of birth, the CVI diagnoses of these authors were not gained until ages 18, 16, and 12 (Nai, Duesing, and Lane-Karnas respectively), and until identification of CVI, these authors, their families, and their healthcare providers had no awareness of CVI.

Nai shares their experience, “Starting at age 12, I was diagnosed with strabismus along all three axes: A-pattern exotropia, heterotropia, and incyclotropia. I was also diagnosed with several oculomotor conditions that are often seen in CVI patients: both accommodative and convergence insufficiencies with a very low AC/A ratio, ciliary spasms, and dysfunction of saccadic, smooth pursuit, and fixation eye movements. At the time, my oculomotor conditions were not understood as potential indicators of an underlying neurological visual disability. Nor was the history of adverse events to my visual development tied to my clinical presentation at the time: being born 3 months premature, incubated for a full year, malnourished in the orphanage, subjected to ongoing deliberate head trauma that was intentionally covered up, and significant visual deprivation due to uncorrected high hyperopia until my first cycloplegic eye exam at the age of 12. Additionally, I had not yet been diagnosed with autism or my genetic disorder, which both show higher rates of sensory processing issues. It was not until I had my first accidental head trauma at 18 due to tripping on a large pile of trash I did not see while walking down a steep sidewalk and being thrust head-first into a brick wall that I was finally diagnosed with cortical blindness based on my MRI. It was only once I studied CVI as a blindness professional that I recognized my lived experiences in descriptions of CVI traits such as visual agnosia, simultanagnosia, and near-total lack of visual imagery. Finally, it is not until discussing my case in detail with CVI-literate eye doctors and researchers that I was able to re-analyze and retroactively understand my CVI holistically. It has only been now, as a blindness professional, that retrospectively, I realize that before I was diagnosed at age 18, I was forced to operate with much less usable vision than many of my ocularly-blind clients who easily qualified as legally blind and benefited from blind services. It was blindness skills that empowered and freed me, not vision therapy or being forced to try to ‘see’.”

Duesing discovered his CVI at the age of 15. It took 15 months, countless doctor’s appointments, and two research studies before he finally received his comprehensive CVI diagnosis. This paper published by Pamir et al. (2021) was the first scientifically documented instance of an individual with CVI, Sebastian Duesing, using verbal mediation to process his vision. In the paper, fMRI data revealed that Duesing can see or not see with his eyes wide open–at will. The Pamir paper describes how Duesing has no conscious perception of sight when he is not using verbal mediation. He spends most of his waking hours fully blind unless he is actively using his extremely limited vision to do a visual task, such as reading or working on an art project. Duesing has normal acuity and severe CVI. He was diagnosed with CVI, including prosopagnosia, object agnosia, topographical agnosia, and simultanagnosia at the New England Eye Low Vision Clinic at the Perkins School for the Blind. The only things that Duesing can see and recognize like a neurotypically sighted person can are words, letters, numbers, and simple shapes. Thus, Duesing always had visual access to numeracy and literacy because he can visually recognize symbols, even though he cannot see and recognize faces, places, objects, or biological forms like a typically sighted person. Duesing was born full term and his CVI is caused by brain damage due to an adverse birth experience. His parents were told that he had a 9 on his APGAR score and the parents were led to believe that he was perfectly healthy. Duesing had all normal developmental milestones and was identified as extremely intellectually gifted as a kindergartner, 2 years before the gifted program at his school began. Duesing developed minor acuity problems when he was about 8 years old. He began wearing glasses at that time and was seen by an eye care practitioner every year after. At no time before the Duesing family discovered his CVI at age 15 did any of Duesing’s medical, educational, or eye care professionals raise any concerns about his vision.

Lane-Karnas was diagnosed with convergence and accommodative insufficiency by their optometrist at age 11, then diagnosed with CVI at age 12 at New England Eye Low Vision Clinic at Perkins, with follow up at Boston Children’s Hospital. Diagnoses include simultanagnosia, prosopagnosia, optic ataxia, visual agnosias (letter, form, and topographic), and oscillopsia. All of Lane-Karnas’ pediatrician eye screenings had been normal and their acuity is normal. Professionals consulted throughout their childhood never suggested Lane-Karnas’ difficulties could be related to impaired vision. Their providers’ best understanding is that their CVI is attributed to birth history, which includes oxygen loss.

## Sensory substitution and augmentation strategies self-developed during childhood

An undiagnosed young child with CVI has no way to ascertain that their visual perception of the world is different from others (Chokron et al., 2021). As young children, these authors who have CVI were unable to make sense of a visually unfamiliar world without appropriate non-visual skills training because they could not see typically. In response, starting in early childhood, Duesing (Pamir et al., 2021), Lane-Karnas, and Nai all self-developed sensory substitution and augmentation strategies to interpret the world non-visually. We describe sensory augmentation as the creation of a novel sense or enhancing an existing one (e.g.: echolocation as creating a novel skill; increasing our sensitivity to sound frequencies as enhancement of an existing one) and sensory substitution as the use of an alternative intact sense for an impaired sense (e.g.: the use of a non-visual alternative such as hearing or touch as a means of obtaining information from the environment). For example, using touch to feel the dots in order to read braille when the written letters cannot be seen is an example of substituting the sense of touch for the sense of vision. Sensory substitution and augmentation are often closely related. Both could also be delivered or assisted with devices. These three authors’ use of non-visual coping techniques extended beyond the traditional “five senses,” using proprioceptive, haptic, and kinesthetic senses. The proprioceptive sense is the internal knowledge of the position and movement of the body in space. Haptic memory relates to the sense of touch, and kinesthetic sense is muscle memory–the ability to sense the position and movement of the body. Duesing, Lane-Karnas, and Nai all have a need for movement and touch to develop proprioceptive, haptic, and kinesthetic memories in the absence of, or severely reduced, ability to form visual memories.

People who have CVI often have difficulty visually recognizing faces, objects, environments, forms, and letters (Farivar, 2009). In addition to these visual agnosias, Duesing, Lane-Karnas, and Nai experience a lack of visual imagery in the mind’s eye. They all use multisensory, semantic, and verbal compensation strategies to substitute for a lack of visual recognition and visual memory. While there is debate in psychology and neuroscience literature about the interplay of visual recognition, visual imagery, and visual memory (Dulin et al., 2008), these three CVIers articulate lived experience of how they cope with these visual deficits. According to Nai, “I used semantic buttressing (processing and retaining a nonlinguistic symbol through its linguistic meaning) to sidestep my inability to visualize or form mental images. For example, I passed visual memory tests by encoding a series of shapes by the sounds they represent in various writing systems, since I have never been able to hold nonlinguistic information in my mind’s eye or visual memory.” Similarly, Duesing uses verbal memory to substitute for lack of visual memory: “I simulate recalling visual imagery by memorizing verbal descriptions of the things I see.” For example, his cat’s characteristics are orange/stripes/yellow eyes. When Duesing thinks those words to himself, he gets a momentary glimpse of what his cat looks like. This is a momentary flash of an image with no retention in visual memory. Duesing says, “I get an idea of it.” Duesing continues, “I also recall motions or gestures made by people or animals as kinesthetic memories, remembering the movement via a mental construct of the feeling of performing that movement.” Duesing uses an enormous, memorized taxonomy of verbal descriptors to identify people, places, and things. Duesing says, “As a young child, drawing helped me practice the skill of identification by salient characteristics, for example, by repeatedly drawing, painting, coloring, and sculpting characters I like. I learned to memorize the features that made them distinguishable. The same skills that allowed me to replicate images of those characters helped me to match people to the characteristics by which I knew them in real life.” Duesing spent thousands of hours of his childhood drawing, painting, and sculpting beginning in late infancy.

Lane-Karnas expands on the challenge posed by lack of visual mental imagery in combination with visual agnosias, describing how it impacts visual memory: “I cannot make visual memories except for colors, and I cannot visually recognize faces, places, objects, or letters, so every time I see something, it is like seeing it for the first time. But I have to remember things, and I can still make memories, so I’ve learned to leverage my other senses to make non-visual memories of visual things. I will use tactile memories of texture, and shape, the imagined feeling of tracing it with my fingers, a distinctive smell, any connected storylines or emotions, and color. My non-visual memory of a chicken consists of the feel of the texture of its feathers; the shape of a chicken in my arms; the specific movement their head makes that I hold as a haptic memory. Then I pull up many storylines of interactions I’ve had with them, and information I know about them.”

Because Duesing, Lane-Karnas, and Nai live with topographical agnosia (impaired to no ability to navigate using visual recognition and visual memory of landmarks and routes) they each self-developed haptic and proprioceptive methods of understanding and mapping familiar environments. Duesing explains, “I depend on proprioception-based memories to navigate familiar spaces. Like the way that others can visualize the layout of a room, I have non-visual memories of the physical feeling of moving around in a space, from which I form image-less ‘maps’ of the positions of walls, doors, furniture, and other objects after I encounter them or maneuver around them physically.”

Nai uses kinesthetic logging to memorize information, which they describe as memorizing environmental information based on how it feels in the muscles, joints, and proprioceptive nerve spindles. Nai states, “The information ‘sticks’ more effectively that way, such as by having spatial information mapped by touch on my back; or repeating instructions to myself in tactile sign language; or by walking routes a few times to encode the proprioceptive information into my nerve spindles. It is helpful to have others spatially map things on my back, especially for new routes or any geographical information, but I also map on my own arm or thighs when processing verbal information too, to better imagine whatever is being described, especially if the spoken content includes verbal step-by-step instructions. I learned the back mapping technique directly from the North American DeafBlind community’s emerging tactual language, called ProTactile. It involves the whole body. Grammar is both spatial and kinesthetic. You sign hand-over-hand, or tactile. I was first introduced to it in 2013 at a workshop at Gallaudet University, and I continue to use it throughout my college education and career. I also use it successfully with in-person CVI students now.”

Lane-Karnas identifies with the haptic and proprioceptive methods of navigating described by Duesing and Nai, depending on the environment: “When I am navigating familiar spaces with my vision, I use mainly a strategy like Duesing described, avoiding obstacles by remembering exactly how it feels in my body to walk around them. When I am navigating unfamiliar spaces or using my cane, I use a strategy more similar to Nai’s, where I imagine drawing a map of my walking paths on my own body and then add tactile details that my cane is giving me, in lieu of a spatial–visual memory.”

Severe visual perception deficits with no awareness of visual impairment can cause trauma. Lane-Karnas explains, “All these things I was doing, I thought were normal, I was unaware other people did not do these things. I had no explanation as to why everything was so much harder and more stressful for me, or why I was constantly more tired, angry, and sad than my friends.” Duesing states, “I always knew that other people were much less freaked out about going places than I was, but I had no idea why.” Nai adds about their own traumatic childhood experience with going undiagnosed, “Since any complaints about being unable to see were met with severe punishments, I had no choice but to develop my coping strategies entirely non-consciously. Being undiagnosed meant I did not have the context or framing to even think of myself as having a legitimate form of blindness. It was only at age 18, when I qualified for services, that I was finally able to consciously think about the non-visual skills I had spent a lifetime developing.”

## Print reading modifications and CVI

Fluent reading ability is crucial to success in society academically, socially, and functionally. Prior to CVI diagnosis, both Lane-Karnas and Nai had severe visual perceptual problems with reading print despite normal visual acuity. Lane-Karnas struggled with reading print and by 12 years old had materials extremely modified by their homeschooling parent in the following ways: limit of 5–15 min of daily print engagement, use of a tinted typoscope, reduced complexity, increased spacing, blue backlit screen, specific sans serif font, and total absence of distraction. Lane-Karnas notes that this “never made a substantial enough difference for me to be at a functional literacy point where I could live a successful life. I was using every other means at my disposal to try to remember something I could not make a visual memory of. I developed touch-memories for the outside shapes of words, by imagining tracing the exterior shape, which I could not see inside of. I was imagining trying to feel the outside of the word, and then using context clues and prediction as well as storylines in my own head to figure out what was going on. And it was practically impossible to maintain because I was holding it in my short-term memory. I was not able to get to a functional reading point; these strategies were not making me literate and were fatiguing to an extreme extent.”

Duesing prefers a 10-point font because he has simultanagnosia, and the small size of the font allows him to see two or three letters at a time. Duesing’s clinically measured, “detection” visual field is full, however his functional field is markedly constricted. He has only a tiny patch of acuity in the center of his visual field where the only things he can recognize like a typically sighted person are words, letters, numbers, and simple shapes. The surrounding area of his visual field is like a dense, colorful fog – a mostly useless blur of light, color, and motion. “Additional print reading modification beyond the requirement of a 10-point font has been largely unnecessary for me. Print reading is one of the relatively few visual tasks I can do easily and comfortably.”

Without diagnosis of CVI in childhood, Nai lacked alternatives to print and suffered. “Trying to look at print was like trying to remain perfectly still and motionless on a boat in the middle of the sea. It made me nauseous and dizzy and exhausted beyond words. This constant sensation of a rocking boat threw a wrench into any hope of comprehension. The motion of the text made it so hard to see the letters that all of my attention was focused on trying to decode each individual letter. I had difficulty visually discerning similar shapes, such as an a from an e, an r from an n, etc. I was so drained from trying to see the letters that I could not follow the story. By the time I got to the bottom of the page, I would not know what the top said. There was no brain space left for tracking the story. All of it got used up on seeing. Looking at the letters. Piecing them together. Trying to follow and track the lines….I would try and try, and I just could not anymore. When I hit that wall, I resorted to self-harm.”

## Vision therapy and CVI

Before CVI diagnosis, Duesing, Lane-Karnas, and Nai undertook vision therapy administered by licensed optometrists in an attempt to improve/cure functional vision through improving oculomotor function and focus using eye exercises. Several licensed optometrists claimed that they could cure Duesing’s CVI and specifically stated that they could cure his visual agnosias and simultanagnosia, which is, of course, impossible. Vision therapy had no effect on his CVI. He did experience an improvement in his convergence insufficiency, an oculomotor problem that developed from a concussion unrelated to his CVI that occurred during his sophomore year of high school. Duesing reflects, “Vision therapy had no effect on my CVI symptoms; it was a frustrating and exhausting experience with no significant benefit.”

Nai remembers, “Vision therapy addressed the oculomotor conditions I had that accompanied CVI but did not improve any of my visual processing issues, as my eye care team was unaware of this deeper layer of visual disability at the time. I developed extreme awareness and conscious control of my intra- and extraocular muscles and over-compensated for accommodation/convergence insufficiency by contracting these muscles hard in short spurts. When I tried my best to approximate horizontal alignment/convergence, my vertical and torsional deviations would skyrocket and trigger hours of extraocular eye muscle cramps and accommodative spasms, which were extremely painful and uncomfortable.”

Lane-Karnas’s vision therapy was undertaken to address accommodative and convergence insufficiency. After vision therapy, they still could not move their eyes while balancing on one foot, had difficulty talking and moving their eyes, and could not keep print in focus to read a paragraph. They comment, “Like Nai and Duesing, vision therapy did not help my CVI. It did not solve my problems with visual recognition and chronic visual fatigue, among other CVI challenges, and it left me exhausted and incapacitated.” Thirty minutes of vision therapy and 5–20 min of vision-based academic tasks daily resulted in a state of chronic visual fatigue and nervous system exhaustion that accumulated into long-term burnout and could not be relieved with rest.

## Sensory substitution and augmentation through non-visual skill training

After a CVI diagnosis, Duesing, Lane-Karnas, and Nai sought training in and currently utilize various non-visual skills and tools, including braille, occupational therapy for navigation, orientation and mobility with a white cane, and access technology. Nai uses a white cane and both text-to-speech software and braille displays on multiple devices (phone and both work-issued and personal laptops) on a daily basis in their professional, advocacy, and personal life. Nai says, “I learned these skills through blind rehab services after my acuity and other low-order processes [visual acuity, visual field, color, and contrast] plummeted as an adult after my accidental head trauma, allowing me to qualify as legally blind. I wish I had had these same strategies when I had the higher-order visual processing issues with perfect acuity.”

Prior to Duesing’s discovery of his CVI, Duesing could not drive, independently navigate on foot, or travel by public transportation. He therefore had no way to safely go to college, begin a career post high school, or live independently. In 2017, Duesing discovered that he had CVI at the age of 15 when he was a sophomore in high school. At that time, no one on Duesing’s educational team had ever heard of CVI or received any training in how to educate or teach orientation and mobility (O&M) skills to a person with CVI. Because Duesing was unsuccessful in accessing those life-saving services from his school O&M instructor, and because the family was unsuccessful at finding a private O&M instructor, and because he had only 2 years before he planned to go to college, the Duesing family had to think creatively to find other solutions.

For Duesing the solution to not receiving a timely CVI diagnosis and access to O&M was privately sourced occupational therapy (OT) for navigation, which insurance paid for, combined with traditional O&M at a non-profit institution which happened more than a year later. For Duesing, OT and O&M were complimentary: he received 3 days per week of OT instruction in navigation over a period of 4 months. In OT, Duesing learned how the address system is organized in a major city; how to use Google Maps on his phone to navigate a major metropolitan city using public transit and on foot, which are skills he could never have received from his suburban school district without missing upwards of 5 h per day of instructional time. Later, Duesing was able to attend a private, 1 week O&M “boot camp” at a guide dog training facility where Duesing received instruction on traveling with a white cane and crossing busy intersections using only his non-visual senses. Duesing notes, “Occupational therapy for navigation and O&M training had a massive impact on my ability to successfully and confidently navigate the world. I do not use a white cane most of the time, but I carry it with me as a back-up, and I am glad to have the option to use it when I want or need it. It taught me how to effectively synthesize the visual skills I do have with a variety of non-visual skills.”

Duesing, a professional ontologist, was a high-performing academic student. He has always been able to access print visually. He says, “I wish that I had the opportunity to learn braille when I was younger, as I think it would have been useful to have a non-visual reading method as an alternate option so that I could engage with text in non-visual ways when I’m low on energy to use for vision. Though I could still learn braille now, I wish I would have had the opportunity to develop fluency with it as a child” (Silverman and Bell, 2018).

After Lane-Karnas was diagnosed with CVI at 12, they began braille instruction which improved literacy and math understanding and retention. Lane-Karnas writes, “Because visual information is so draining and hard to remember, I have learned non-visual ways to get necessary information I use every day, such as braille, a screen reader, and a long white cane. These tools greatly reduce the amount of visual fatigue in my life, leaving me more able to be happy, and more able to do visual things that I choose to do, like art. Reading print as a 12-year-old I would continually meltdown, cry, and be chronically dysregulated. Now, using braille, access tech, and non-visual learning, I can attend instruction in homeschool, classes, independent study, and tutoring that can range from 30 min to 2 h multiple times a day. I will still be fatigued but am able to quickly recover and pace my day such that I am able to engage in hobbies, time with friends, and family activities. I am also better able to retain and engage with the non-visual information.”

## Optimizing conceptual understanding using sensory substitution and augmentation

Duesing, Lane-Karnas, and Nai describe how their vision as a sensory input is often replaced or informed by other senses. Their use of non-visual senses is consistently misinterpreted by sighted observers, who presume a task is being done visually. Nai explains: “My brain is naturally hyper-kinesthetic to the point my physical therapists have regularly commented I have exceptionally sensitive interoception (internal sensations of the body). I have relied heavily on my interoception as a substitution for visually guided reach throughout my whole life. For example, I know where to reach for the knife on the rack by how the angles feel in my joints; it appears to the outside observer like I am focusing on a visual target, but I am actually guided by the sensation in my muscles, tendons, and bones. I also learned American Sign Language by feeling angles in my fingers, hands, wrists, and arms because I could not see the signs.”

Lane-Karnas is a visual artist and since late infancy has spent hours a day drawing, painting, or sculpting. They have no visual memory of anything they have ever seen or created, and due to simultanagnosia, have never seen their own art in its entirety. Lane-Karnas references using verbal, kinesthetic, and touch modalities to support their art. “When I want to learn how to draw a new thing, it takes a lot of intentional practice, repetition, and visually studying the thing before I can consistently draw it. I’ll add words and storylines to all the elements of the thing so I can remember it in enough detail to draw it accurately, and then break those elements down into shapes and encode the movement of drawing the shapes into my hand so I can consistently draw it the same. This process has the added benefit of really strengthening my memory of the figure of the thing and giving me a firmer understanding of what it is. It also helps to identify common visual patterns and expectations which I can use to more easily visually identify the thing.” As a younger child Lane-Karnas sought any representations of the human form that they could touch: dolls, posable figures, sculptures, mannequins, and bones, spending endless hours posing and playing with them, drawing the human form, and deconstructing clothing to learn how it is constructed around the body. “Having a tangible, tactile interaction with something helps me to cement what the thing even is; it lets me become clearer on how it is constructed, built, or functions, and have the knowledge to much more accurately reflect what it is through visual arts.”

Duesing’s internal experience of tasks presumed to be entirely visual are not at all so: “Activities that other people often think of as inherently visual tasks, like playing video games or drawing, are multisensory experiences to me. Muscle memory and proprioceptive understandings of shapes and patterns fill the void left by my absence of visual memory and my visual processing. Though the outcomes of those so-called visual tasks are approximately the same for me and those who do perform them visually, the processes by which we reach those outcomes are very different.” Duesing, who has object agnosia, uses haptic memory for tasks like distinguishing ordinary objects that he has frequently held in his hands. “When I am looking for a piece of produce in the grocery store, I cannot always rely on everything to be labeled, which is normally my first-line strategy for identifying objects in a store, because I can read visually. If, for instance, I’m looking for an apple, I imagine the feeling of holding an apple in my hand, and I look for things that fit the approximate shape and size that I ‘feel.’ If I look at a pineapple, I know that’s not the item I’m looking for, because, without touching the fruit, I can feel in my hand that it’s too big. If I look at a banana, I know that it’s not what I’m looking for, because I can feel that it is too small, even though I have not touched it. Most things can be ruled out like this. I only lean on looking for more vision-intensive characteristics like texture or color when I cannot rule things out by shape and size, like telling an orange from an apple.”

Like Lane-Karnas, Duesing is also a visual artist, and reiterates, “Things that seem like visual tasks do not have to be. I find that drawing things requires a lot of study, practice, and repetition. Because I have no visual memory of, e.g., people, I cannot rely on a memory bank of experiences of looking at people in order to draw them. I can represent humans fairly accurately in my art because firstly, I’ve spent a lot of time studying the canon of proportions of the human body, and secondly, I use a lot of reference images for pretty much everything I draw in order to get body positioning, lighting, and perspective right. I also have to work with a small area of useful vision, so I find it easiest to work very small during the initial parts of the drawing process in which I’m determining larger-scale features like composition, poses, and proportions. By “working small,” I mean things like doing thumbnail sketches on a small section of the canvas, zoomed far out, as the first step in the drawing process, and trying to get composition/pose/proportions looking good before I worry about representing any detail at all.”

Unlike Duesing and Lane Karnas, Nai found trying to participate in the visual arts to be extremely frustrating. Nai says, “Visual arts were not helpful to me for interpreting the world. It was a torture I endured because of school requirements.” The sensory substitution and augmentation techniques utilized by one CVIer may be entirely inappropriate for another due to the unique challenges presented by CVI and their different life experiences.

## Discussion

Despite advances in describing the manifestations of CVI in academic, research, and clinical literature, the voices of individuals affected by CVI remain largely unheard in peer-reviewed literature. Listening to patients’ lived experiences has been linked to improved healthcare outcomes. Studies by Coulter (2012) and Edbrooke-Childs et al. (2016) show that patient involvement in decision-making leads to better treatment results, increased satisfaction, and more effective care. Systematic reviews by Gleeson et al. (2016) and Bate and Robert (2007) further highlight that incorporating patient feedback into service improvement enhances both clinical outcomes and overall patient wellbeing (Bate and Robert, 2007; Coulter, 2012; Edbrooke-Childs et al., 2016; Gleeson et al., 2016). This is true for children with VI regarding improved healthcare outcomes. Robertson et al. (2021) and Lupón et al. (2018) show that involving families in care decisions enhances emotional and social well-being. Lanza et al. (2024) emphasizes the value of integrated assessments that consider these experiences. Douglas, (2006) demonstrate how understanding the broader impacts of VI leads to more effective support (Douglas, 2006; Lanz, 2024; Lupon, 2018; Robertson et al., 2021). Incorporating these perspectives into care results in better overall outcomes for children with VI.

The significant impact of CVI on patients and their families is just starting to be addressed through the direct voices of CVIers in the literature. The importance of listening to CVIers and their families has been highlighted in semi-structured interviews (Jackel et al., 2010; Falkenberg et al., 2020; Goodenough et al., 2021; McCarty and Light, 2023; Oliver et al., 2023). An approach to visual, emotional, and behavioral needs of the CVI population has been suggested based on a literature review (McDowell, 2023). CVIers have expressed their challenging experiences in support websites (e.g.: CVI Scotland; Perkins School for the Blind). However, until very recently, the direct written experiences of people who have CVI have been missing in the peer-reviewed literature, which has contributed to the existing challenges in underdiagnosis, misdiagnosis, and a lack of CVIer-led intervention methods in the management of CVI. In this paper we demonstrate the value of incorporating direct, self-written perspectives into the published literature in order to advance understanding of CVI.

This paper details self-developed sensory substitution and augmentation strategies, as well as the essential need for non-visual skills training in three CVIers with HVFDs. Multisensory intervention has been used successfully for people who have no residual vision and for people with severe visual acuity and/or visual field impairments (Gori, 2015; Purpura et al., 2017; Morelli et al., 2020). Successful implementation of braille reading has been described in a CVIer with good visual acuity (Lane-Karnas, 2023), and recommended by clinicians (Kran et al., 2019) and educators (Lueck and Dutton, 2015), but it is not routinely available to CVIers as part of the (re)habilitation process, especially when visual field and visual acuity are not impaired to significant levels. In addition, CVIers need to be assessed for functional vision, including their abilities with visually guided behaviors, with particular attention paid to the concerns of the CVIers themselves and/or their families. Clinically administered evaluations of visual function (acuity and field) frequently do not meet criteria for access to non-visual skills training such as braille, O&M, and access technology. The NIH’s emphasis on the importance of an individualized approach for the educational, vocational, recreational, and social needs of CVIers (Chang et al., 2024) is strongly illustrated by the heterogeneous needs of these authors who have CVI.

Lacking typical visual perception, the authors recount how proprioceptive, haptic, and kinesthetic senses are crucial for their ability to form non-visual memories in order to do tasks and to navigate familiar environments. Duesing, Lane-Karnas, and Nai detail the importance of O&M training and OT for navigation to learn to navigate in unfamiliar environments in order to live independently, along with other interventions, to mitigate psychological harm from years spent being blind in a world without non-visual skills. CVI visual impairments that prevented fluent print reading, such as letter agnosia and oculomotor issues, and undergoing ineffective vision therapy, further emphasize the importance of developing non-visual skills, such as with braille and access technology. The authors explain how non-visual learning methods effectively improved their academic, social, and personal learning that had been impossible to achieve using visual means.

In this paper, individuals with CVI describe their experiences with vision therapy (eye exercises intended to promote better use of both eyes together as a team). Duesing most likely benefited from vision therapy for his post-concussion convergence insufficiency (Scheiman et al., 2017) during his teenage years. However, the paradigms of behavioral vision therapy (VT) instituted for other symptoms and signs, as experienced by all three authors with CVI, were ineffective in addressing their visual perceptual and oculomotor challenges. The authors underwent VT during childhood during the years when the literature questioned its efficacy as a treatment; reviews of studies on VT for convergence insufficiency had reported minimal benefits (Rawstron et al., 2005; Barrett, 2009), while Ciuffreda (2002) suggested convincing lab-based models for background evidence and recommended further research. At no point in their treatment were these controversies from the published literature research discussed with these three authors who underwent vision therapy, or their families. In later years, Scheiman (2018) provided a comprehensive review toward establishing an evidence-based foundation for vision therapy, particularly in the treatment of convergence insufficiency, while also advocating for continued scientific inquiry to strengthen the field’s credibility in areas where the evidence is still mixed or lacking. A Cochrane meta-analysis reported with moderate certainty evidence that pediatric patients with convergence insufficiency are 5 times more likely to show fewer symptoms and better outcomes after office-based vision therapy with home reinforcement compared to placebo therapy (Scheiman et al., 2020). However, this review did not address the efficacy of vision therapy in individuals with CVI. Recently, Flaherty et al. (2024) critically assessed behavioral vision therapy for children with reading difficulties, including dyslexia, noting limited evidence supporting its effectiveness. While some improvements in visual processing were observed, they emphasize the need for more rigorous, evidence-based research to confirm its impact on reading outcomes. None of the studies in the past or recently, had children with CVI. Research into the causes of commonly occurring near focusing and eye movement difficulties in CVI is ongoing, with no proven cause or treatment. While VT may be beneficial for some disorders, the authors recommend prospective controlled trials to determine its efficacy for CVI, as current evidence does not support its clinical use in this population.

In this article, important and unique information is presented by CVIers themselves about self-developed compensatory strategies that were developed without the knowledge of the parents. For example, for two of the authors of this paper, their intense, lifelong interest in the arts helped them to develop a richer understanding of the visual world without any parental or educational awareness of what was driving these CVIers’ passionate pursuit of the arts. There is increasing evidence that effective technology and interventions are best developed and implemented with the involvement of the blind and visually impaired (Conradie et al., 2015; Magnusson et al., 2018) and should not be led only by sighted observers or by simulations of visual impairment (Qiu et al., 2024). We provide information which strongly suggests that CVIers and their families should be involved in all aspects of research to improve clinical understanding of CVI and its impacts on education and (re)habilitation. This collaborative effort is essential to developing and implementing a multisensory, integrative approach that is founded on the autonomy and choice of the affected CVIer based on their personal interests and abilities rather than on inaccurate assumptions made by typically sighted outside observers.

Currently, there are at least 180,449 individuals in the US aged 0–22 with diagnosed or likely CVI, with a diagnostic rate under 14% (White Paper CVI Perkins). Additionally, there is a lack of evidence-based, scientifically valid training in CVI for low vision professionals (Harpster et al., 2022; Lueck et al., 2023), as well as a significant shortage of teachers of the visually impaired (TVIs) and O&M specialists (Schles and Chastain, 2023; Randles, 2020). This shortage, made worse with few with expertise in CVI, is affecting service delivery for young individuals diagnosed with CVI. As the number of CVI diagnoses increases, the demand for low vision services, coupled with the limited availability of trained professionals, underscores the need for solutions to address the growing gap in service provision. For example, while the use of OT for teaching independent travel in combination with O&M training with a white cane for CVIers may be novel in the United States, the NIH has recently affirmed the importance of cross-specialty cooperation due to the interdisciplinary effects on individuals with CVI (Chang et al., 2024). Furthermore, the current definition of legal blindness, which relies solely on measures of acuity and visual field, frequently limits the ability of CVIers of all ages to access non-visual learning and (re)habilitation services (Kran et al., 2019). As more people with CVI are diagnosed, the large numbers of people needing low vision services and the lack of providers is cause for concern and raises the need for creative solutions to help this overlooked and vulnerable population. According to the NIH, there is a lack of adequate transition planning to adult care for the majority of people with any form of vision impairment and providing necessary and often life-saving support for CVIers across their lifetimes is of the utmost importance (Chang et al., 2024). For these reasons, it is especially important to consider how late diagnosed individuals with CVI who are aging out, or have already aged out of public school, will access non-visual skills training that are typically accessed by ocularly blind children in the school setting.

## Conclusion

Every person who has CVI has a unique experience of their own functional vision (Lueck and Dutton, 2015). Having firmly established the prevalence of HVFDs in CVI at all levels of acuity loss (Ortibus et al., 2011; Chandna et al., 2021a, 2021b; Sakki et al., 2021), the need is great in the medical and education fields to understand common visual perceptual differences in their HVFD spectrum and severity as a heterogenous group as well as for each individual. Direct dialogue with CVIers and their parents uncovers rich details of shared and disparate experiences living with CVI; and of sensory augmentation and substitution that improves understanding of the world in the absence of typical vision. Sensory substitution and augmentation techniques employed by the three blind authors of this paper are invisible to the typically sighted observers and are therefore impossible to identify through observation of behaviors alone, illustrating the essential need for dialogue between CVIers themselves and parents, medical, and scientific professionals. As CVIers begin to share their firsthand experiences with sensory substitution and augmentation techniques used to understand the visual world, consideration must be given to how these stories can be used to help accurately describe the CVI experience; plan research on effective multisensory educational and therapeutic interventions; achieve an accurate diagnosis; and ensure individualized, accessible education and habilitative services.

## Data Availability

The original contributions presented in the study are included in the article/supplementary material, further inquiries can be directed to the corresponding author/s.

## Appendix

Access technology: When AT is mentioned, it is meant to include AAC devices, braille displays/notetakers, and all means of audio-based tools such as screen readers, dictation software, audio books.

Agnosia, visual: decreased or absent ability to visually recognize or identify faces (prosopagnosia), shapes, objects, symbols, environments (topographical agnosia).

CVIer: The term “CVIer” was first coined by one of the authors, Nai, and its use has become accepted and spread in professional and informal settings over the last several years. This reflects and respects our community’s growing sense of self and acceptance, and our understanding of belonging within the larger neurodiversity paradigm.

Kinesthetic logging (term introduced by Nai): memorizing environmental information based on how it feels in the muscles, joints, and proprioceptive nerve spindles. It is different from touch because it is not the sensation of the atmosphere on the surface of the skin, but the feeling inside the body relative to the environment.

Non-visual skills: skills such as braille, O&M, access technology, etc. (or as blindness rehabilitation professionals call” blind skills”).

Semantic buttressing (term introduced by Nai): processing and retaining a nonlinguistic symbol through its linguistic meaning. For example, interpreting a circle as the letter O, a triangle as the capital Greek letter delta *Δ*, and a square as the Chinese character 口 (kǒu, “mouth”).

Sensory augmentation: creating a novel sense or enhancing an existing one (e.g.: echolocation as creating a novel skill; increasing our sensitivity to sound frequencies as enhancement of an existing one).

Sensory substitution: the use of an alternative intact sense for an impaired sense (e.g.: the use of a non-visual alternative such as hearing or touch as a means of obtaining information from the environment). For example, using touch to feel the dots in order to read braille when the written letters cannot be seen is an example of substituting the sense of touch for the sense of vision. Sensory substitution and augmentation are often closely related. Both could also be delivered or assisted with devices.

Typoscope: a tool that obscures all lines of text on a page except for the one line the reader is focusing on.
